# Classification of transient epileptic amnesia attacks: Two types of amnestic seizures, the pure amnesia type and partial amnesia type

**DOI:** 10.1002/pcn5.114

**Published:** 2023-06-23

**Authors:** Katsuyuki Ukai, Masumi Ito, Masako Watanabe

**Affiliations:** ^1^ Department of Psychogeriatrics Kamiiida Daiichi General Hospital Nagoya Japan; ^2^ Department of Psychiatry Nagoya University Graduate School of Medicine Nagoya Japan; ^3^ Jozen Neuro Clinic Sapporo Japan; ^4^ Shinjuku Neuro Clinic Tokyo Japan

**Keywords:** accelerated long‐term forgetting, classification, pure amnesia, topographical amnesia, transient epileptic amnesia

## Abstract

**Aim:**

Transient epileptic amnesia (TEA) is a type of mesial temporal lobe epilepsy characterized by recurrent amnesia attacks. In 1998, Zeman et al. proposed the following diagnostic criteria for TEA: (1) recurrent, witnessed episodes of amnesia (TEA attacks); (2) other cognitive functions remain intact during attacks; and (3) evidence of epilepsy. It was also reported that patients with TEA often demonstrate two other types of memory symptoms: accelerated long‐term forgetting (ALF) and autobiographical amnesia (AbA). Both ALF and AbA are persistent memory disorders, but transient epileptic seizures are not.

**Methods:**

We encountered two cases of TEA associated with two types of amnesia attacks. Therefore, we reviewed TEA cases in the literature to clarify the type of TEA attacks that occurred.

**Results:**

Based on the extracted TEA cases, including our two cases, we found that there are two main types of TEA attacks, and we discussed their clinical features.

**Conclusion:**

We propose two main types of TEA attacks; that is, pure amnesia‐type and partial amnesia‐type seizures. Furthermore, we also propose that topographical amnesia mainly manifests as a type of amnesia attack, rather than as a chronic memory disturbance, such as ALF or AbA.

## INTRODUCTION

Transient epileptic amnesia (TEA) is a type of mesial temporal lobe epilepsy characterized by recurrent amnesia attacks. In the 1990s, Hodges and Warlow^1^, Kapur,^2^ and Zeman et al.^3^ described the clinical features of TEA. Zeman et al. proposed the following diagnostic criteria for TEA: (1) recurrent, witnessed episodes of amnesia (TEA attacks); (2) other cognitive functions remain intact during attacks; and (3) evidence of epilepsy. Such evidence can be provided by (a) electroencephalography (EEG), (b) the co‐occurrence of other types of seizure, or (c) a clear response to antiepileptic drugs.^3^ TEA attacks typically last for minutes to hours. Patients with TEA often demonstrate two other types of memory symptoms: accelerated long‐term forgetting (ALF) and autobiographical amnesia (AbA).^4^, ^5^ Both ALF and AbA are persistent memory disorders, but transient epileptic seizures are not.

Here, we present two cases of TEA, in which the patients showed two types of amnesia attacks; that is, pure amnestic seizures and topographical amnesia. Furthermore, we reviewed the TEA cases described in the literature to clarify the features of TEA attacks. In addition, we show that topographical amnesia often manifests as a type of amnesia attack, rather than as a chronic memory disturbance, such as ALF or AbA.

## METHODS

Informed consent to publish the patients’ clinical information was obtained from the patients and their families. Minor modifications to the data, which did not interfere with the study's findings, were made to preserve the patients’ anonymity. The detailed course of Case A has been previously reported.^6^ The study was approved by the ethics committee of Kamiiida Daiichi General Hospital (Nagoya, Japan).

## RESULTS (CASE PRESENTATION)

### Case A (cited from Ukai et al.,^6^ partially modified and simplified)

A male in his early 60s attended our memory clinic with his wife in 200X. He said that his memory had become faulty about 4 years earlier, and he had experienced many problems at his workplace because of his forgetfulness. He also stated that he had forgotten many events, such as traveling with his wife a few months earlier, undergoing surgery about 2 years earlier, and the wedding ceremony of his daughter about 11 years earlier. EEG was performed, and sharp waves were detected, which seemed to originate independently in the left and right temporal areas. The administration of levetiracetam was commenced, resulting in an improvement in the patient's anterograde amnesia; that is, ALF.

In 200X + 4, the ALF reappeared, and a pure amnesia attack was observed for the first time. One day in the morning, he and his wife went to a barbershop, where they met a close friend and enjoyed an interesting conversation. However, in the afternoon he asked his wife what he had done in the morning. His wife was very surprised and asked him some questions, but he did not remember going to the barbershop, having his hair cut, or meeting and talking to their friend. She said that his behavior had been quite normal that morning. The dose of levetiracetam was increased, which resolved his amnesia attack.

More than 2 years after that (in 200X + 6), a second amnesia attack occurred. The patient and his wife went to a hot spring with their friends in his car, which he drove. On his way back home in the afternoon, he took his friends home and then returned to his home. After that, he went to a gym alone by car, before returning home about an hour later. Then, he ate dinner with his wife. After dinner, he was sitting on the couch in the living room, looking at a calendar for a while, and he asked his wife what they had done in the afternoon. According to his wife, he remembered going to the hot spring with their friends and then taking them home. However, he did not remember anything during the few hours between returning home and having dinner. During this period, he was able to drive his car well and engage in meaningful conversation.

### Case B

A male in his late 70s attended our memory clinic with his wife in 200X. He complained that his memory had become faulty about 2 years earlier and that he sometimes lost his way in places that he had known well, for example, near the subway station for his office, to which he had been commuting for decades. During these episodes, he would suddenly lose his way, but was able to do his work well after arriving at his office. He also stated that he sometimes became unable to recall what he had done for a few hours. He always recognized these episodes well afterwards. His wife also sometimes witnessed his episodes of amnesia. For example, (1) while driving his car to a place that they had often visited he said, “We are going straight here, right?” She was surprised and said to him, “No, turn left! What's wrong with you?” (2) One evening, he came back home from his workplace and said to her, “What did I do today? I cannot recall it.”

Cranial MRI did not reveal any abnormalities, except for relatively prominent high‐intensity lesions of the cerebral white matter on fluid‐attenuated inversion recovery imaging. Although his EEG showed no evident paroxysmal activity, the administration of lacosamide was commenced under a strong suspicion of TEA, resulting in the complete disappearance of the patient's amnesia attacks. Based on these clinical findings, we diagnosed him as having pure amnesia seizures and topographical amnesia attacks; that is, TEA attacks.

## DISCUSSION

We reviewed articles regarding TEA, including those by Palmini et al. (1992), Kapur (1993), Zeman et al. (1998), Mendes (2001), Manes et al. (2005), Gallassi (2006), Butler et al. (2007), Hornberger et al. (2010), Mosbah et al. (2014), Cretin et al. (2014), Savage et al. (2016, 2018, 2022), Burkholder et al. (2019), and Shiozaki (2019, 2020).^1^, ^2^, ^3^, ^4^, ^5^, ^7^, ^8^, ^9^, ^10^, ^11^, ^12^, ^13^, ^14^, ^15^, ^16^, ^17^, ^18^, ^19^, ^20^ However, there were few cases in which TEA attacks were described in detail. We examined 23 TEA cases, including the two cases we described above (Table 1). Based on these cases, we classified TEA attacks into the following two main types: the “pure amnesia type” and “partial amnesia type.” The partial amnesia type can be further classified into several subtypes, including the “topographical amnesia type.”

**Table 1:** Examples of TEA attacks described in representative reports, including our two cases.

1st Author	Case	Age (years)	Sex	Examples of TEA attacks described in the paper	Type of amnesia
Palmini^7^	1	30	F	She had no recollection whatsoever of her conversation.	Pure
	2	36	F	She suddenly realized that she had a blank in her memory.	Pure
	3	44	M	He realized that he had no memory of what he had done.	Pure
	4	52	F	She had no recollection of what she had done.	Pure
	5	32	M	He had no recollection of how he had reached the place where he had parked his car.	Pure
Kapur^2^	1	63	F	She forgot the name of a family member.	Partial (family/home)
	2	67	F	She could not recollect having visited her daughter. “I don't know where I am.” She experienced sudden‐onset disorientation regarding where she was and why she was there.	Pure Partial (TopA)
	3	61	F	She could not recognize her room, husband, or children. She forgot what she had just done.	Pure Partial (family/home)
Zeman^3^	1	63	M	He has no recall of events during the attacks. “I am not sure where I am.”	Pure Partial (TopA)
	2	73	M	“What are we doing here?”	(TopA??)
	3	60	M	He developed amnesia regarding recent events, familiar faces, and places.	Partial (family/home and TopA)
Mendes^8^	1	45	M	He asked his colleague what had happened before.	Pure
	2	71	F	She forgot the fact that she had just been to a supermarket.	Pure
	3	74	M	“Where are we now? Why did we come here?” He did not know what they were going to do.	Pure (and TopA also??)
Manes^4^	1	59	F	She experienced disorientation episodes during which she felt muddled about her surroundings.	(TopA??)
Butler^5^	1	58	M	He experienced significant new difficulties navigating around his local area.	(TopA??)
	2	69	F	She experienced loss of memory about events of the past few days.	(Pure??)
	3	55	M	“I've lost it. Where am I? What day is it?”	Partial (date/time and TopA)
Burkholder^17^	1	50	F	She experienced episodes involving an inability to recognize family members.	Partial (family/home)
Shiozaki^18^	1	70s	M	(described in Japanese)	Partial (TopA) (Pure also??)
Shiozaki^19^	1	60s	M	(described in Japanese)	Partial (TopA)
Ukai	A	60s	M	He asked his wife what he had done in the morning.	Pure
	B	70s	M	He sometimes lost his way to places he had known well. “What did I do today?”	Pure Partial (TopA)

Abbreviations: Partial, partial amnesia type; Pure, pure amnesia type; TEA, transient epileptic amnesia; TopA, topographical amnesia type.

### Pure amnesia‐type TEA attacks

In 1992, Palmini et al.^7^ defined “pure amnestic seizures” (PASs) as seizures during which the only clinical manifestation is the patient being unable to retain memory events that had occured during the seizure, despite their other cognitive functions and the ability to interact normally with their physical and social environment being preserved. Most of the patients reported by Palmini were young (Table 1), and their epilepsy may have had organic causes, but typical TEA patients are middle‐aged to elderly at the time of onset, and the causes of their attacks are usually not obvious. Thus, the relationship between PASs and TEA attacks remains unclear, but it is considered that these types of seizures are closely related to each other and have essentially the same pathogenesis. The TEA episodes that occurred in our Cases A and B were very similar to PASs. Thus, we referred to this type of TEA attack as the “pure amnesia type.”

In many of the cases reported in the literature, TEA attacks occurred upon waking. On the other hand, there were also cases in which TEA attacks occurred during daily activities. When pure amnesia‐type attacks occur during activities, patients can behave normally even when they are performing difficult or complex tasks, such as driving, playing golf, or having a conversation. Hence, it is common for none of the people around a patient during an attack to be aware that the patient is experiencing abnormalities. After an attack, patients recognize that they cannot recall what they did for minutes or hours. Some typical examples of pure amnesia‐type attacks are described for our two cases.

### Partial amnesia‐type TEA attacks

A feature of this type of seizure is that during these seizures patients usually find that some or most of their memories have been lost, for example, they cannot remember information about the date/time, their family/home, or familiar places. In addition, during such seizures patients often notice their memory losses and/or are sometimes aware of difficulty remembering new things. In other words, patients are usually able to realize that they are experiencing an abnormality due to memory loss during these seizures. Therefore, repeated questioning of other people is often observed during this type of seizure.^2^

On the other hand, in pure amnesia‐type seizures, neither the patient nor the other people around them recognize that the patient is experiencing abnormalities during the attacks. Thus, repeated questioning is not observed during pure amnesia‐type seizures. Only after the seizure does the patient realize that they do not know what they have done for minutes to hours.

As elements of the patient's memory are lost (partial memory loss), we referred to this type of TEA attack as the “partial amnesia type.” Furthermore, based on the lost elements of memories, it is possible to classify partial amnesia‐type seizures into at least four subtypes; that is, (1) the “topographical amnesia type,” (2) the “date/time amnesia type,” (3) the “family/home amnesia type,” and (4) other types.

During topographical amnesia‐type attacks, patients temporarily lose track of their locations and forget how to get to destinations that should be familiar to them. After they recall their location and/or their intended direction, they usually remember the episodes of amnesia well. Some specific typical examples of topographical amnesia attacks are described for our Case B.

During date/time amnesia‐type attacks, patients temporarily cannot remember the current year, season, month, day, or time. For example, a patient repeatedly asked his wife what day it was. In some cases, the patient could not remember their age. On the other hand, during family/home amnesia‐type attacks, patients temporarily cannot recognize their family members or their own home. For example, a patient woke up and found some strangers (her family members) there and herself in an unknown house (her own house).

Each subtype of partial amnesia‐type attack may occur together with other subtypes of attack, or one subtype may occur independently. On the other hand, pure and partial amnesia‐type seizures cannot occur simultaneously. However, both seizure types can occur independently at different times.

### Topographical amnesia: A seizure or chronic symptom

In general, topographical amnesia is referred to as one of the chronic sequelae associated with an acute stroke or brain hemorrhage. The topographical amnesia associated with TEA (TopA) is also usually described in the literature as a chronic symptom, as are ALF and AbA. TopA was defined as difficulty recollecting the layout of previously familiar environments and/or a failure to recognize previously familiar landmarks and locations.^20^ However, in our Case B, TopA was a transient symptom. Several other similar cases have been reported (Table 1). We speculate that both scenarios are possible. In cases in which TopA is described as a chronic symptom, it may be explained as a consequence of ALF and/or AbA.

### Limitations of this study

This study had several limitations:

(1) In order to classify TEA attacks, 23 TEA cases were examined in this study, but this may not have been sufficient.
(2) The frequencies of each type of TEA attack should be elucidated in the future.
(3) Other subtypes of TEA attacks may exist.

We consider that it is important for clinicians to distinguish among TEA attacks, ALF, and AbA, in order to be able to diagnose and treat these conditions earlier and more accurately.

## CONCLUSIONS

In conclusion, this study revealed the following:

1. Based on our clinical experience and a literature search, we classified TEA attacks into two main types: “pure amnesia type” and “partial amnesia type.”
2. When pure amnesia‐type attacks occur, neither the patient nor the people around them recognizes that the patient is experiencing abnormalities. Only after the seizure is over, do patients realize that they have lost their memories.
3. When partial amnesia‐type attacks occur, patients usually notice that some or most of their memories have been lost, and that it is difficult to remember new things. Hence, repeated questioning of other people with queries such as, “Where am I?”, “What day is it?”, and “Who are you?” is often observed during this type of seizure. Based on the lost elements of memories, we have classified partial amnesia‐type attacks into several subtypes, including TopA.
4. TopA is usually described in the literature as a chronic symptom, as are ALF and AbA. However, we consider that TopA mainly manifests itself as an attack, rather than as a chronic memory disturbance, such as ALF or AbA.

## AUTHOR CONTRIBUTIONS

Katsuyuki Ukai collected and studied the clinical data, wrote the initial draft, and edited the manuscript. Masumi Ito and Masako Watanabe critically reviewed the manuscript and edited the manuscript. All of the authors have approved the final manuscript as submitted.

## CONFLICT OF INTEREST STATEMENT

The authors declare no conflict of interest. Dr. Ukai received speaker's honoraria from Daiichi Sankyo in 2022. Dr. Ito has received speaker's honoraria from Nippon Chemiphar, Eisai, and UCB Japan. Dr. Watanabe has received speaker's honoraria from Otsuka, Eisai, Daiichi Sankyo, and UCB Japan. No grants or other sources of funding were received for this study.

## ETHICS APPROVAL STATEMENT

The study was approved by the ethics committee of Kamiiida Daiichi General Hospital (Nagoya, Japan).

## PATIENT CONSENT STATEMENT

Informed consent to publish the patients’ clinical information was obtained from the patients and their families.

## CLINICAL TRIAL REGISTRATION

N/A.

## Data Availability

On reasonable request, data supporting the findings of this study are available from the corresponding author after approval from the ethics committee.
